# Viewing RNA-seq data on the entire human genome

**DOI:** 10.12688/f1000research.9762.1

**Published:** 2017-04-28

**Authors:** Eric M. Weitz, Lorena Pantano, Jingzhi Zhu, Bennett Upton, Ben Busby

**Affiliations:** 1 National Center for Biotechnology Information, U.S. National Library of Medicine, Bethesda, MD, 20894, USA; 2 Harvard T.H. Chan School of Public Health, Boston, MA, 02115, USA; 3 Koch Institute for Integrative Cancer Research, Massachusetts Institute of Technology, Cambridge, MA, 02139, USA; 4 LSU Shreveport Laboratory for Advanced Biomedical Informatics, Shreveport, LA, 71105, USA

**Keywords:** RNA-seq, ideogram, javascript, next generation sequencing

## Abstract

RNA-Seq Viewer is a web application that enables users to visualize genome-wide expression data from NCBI’s Sequence Read Archive (SRA) and Gene Expression Omnibus (GEO) databases. The application prototype was created by a small team during a three-day hackathon facilitated by NCBI at Brandeis University. The backend data pipeline was developed and deployed on a shared AWS EC2 instance. Source code is available at https://github.com/NCBI-Hackathons/rnaseqview.

## Introduction

Interactive visualizations can yield insights from the deluge of gene expression data brought about by RNA-seq technology. Several genome browsers enable users to see such data conveniently plotted within a single chromosome in a web application ( Broad Institute, 2014; Kent *et al.*, 2002; National Center for Biotechnology Information: Genome Data Viewer (2016)). While such single-chromosome views excel at displaying local features, depicting RNA-seq data across all chromosomes in a genome, i.e. in an ideogram, has the potential to intuitively highlight global patterns of gene expression (such as in Figure 2a in Parker *et al.*, 2016).

In this paper we describe RNA-Seq Viewer, a web application that enables users to visualize genome-wide expression data from the National Center for Biotechnology Information’s (NCBI) Sequence Read Archive (SRA) ( Kodama *et al.*, 2012) and Gene Expression Omnibus (GEO) ( Barrett *et al.*, 2013) databases. The application consists of a backend data pipeline written in Python and a web frontend powered by Ideogram.js, a JavaScript library for chromosome visualization ( Weitz, 2015).

The data pipeline, developed by a small team of software engineers in a three-day NCBI hackathon at Brandeis University, extracts aligned RNA-seq data from SRA or GEO and transforms it into a format used by Ideogram. Ideogram then displays the distribution of genes in chromosome context across the entire human genome and enables users to filter those genes by gene type or expression levels in the given SRA/GEO sample.

## Methods

The primary task of the hackathon was to develop a prototype data pipeline to extract aligned RNA-seq data from SRA, determine genomic coordinates for the sampled genes, and transform the combined result into the JSON format used by Ideogram.js annotation sets. The formatted annotation data was then plugged into a lightly modified example from the Ideogram repository to provide an interactive, faceted search application for exploring genome-wide patterns of gene expression.

Ideogram.js uses JavaScript and SVG to draw chromosomes and associated annotation data in HTML documents. It leverages D3.js, a popular JavaScript visualization library, for data binding, DOM manipulation, and animation ( Bostock *et al.*, 2011). Faceted search in Ideogram is enabled by Crossfilter, a JavaScript library for exploring large multivariate datasets ( Square Inc., 2012). By relying only on JavaScript libraries, HTML and CSS, Ideogram can function entirely in a web browser, with no server-side code required, which simplifies embedding ideograms in a web application.

Annotation data for Ideogram leverages space-efficient data structures and the compact nature of JSON to minimize load time in web pages. For example, the gzip-compressed set of 31,148 human gene feature annotations, including data on expression level and gene type, output by our pipeline for SRA run SRR562646 ( National Center for Biotechnology: Sequence Read Archive Run Browser) is 399 KB in size and takes less than 285 ms to download on an average US Internet connection (14 Mb/s download bandwidth, 50 ms latency) ( Belson *et al.*, 2016) as measured using Chrome Developer Tools ( Basques & Kearney, 2016). Under the same network-throttled conditions using Chrome version 51 on a Mac OS X laptop with a 2.9 GHz Intel Core i5 CPU, the Chrome DevTools Timeline tab reports that an uncached, interactive genome-wide histogram of expression for 31,148 gene features takes Ideogram between 830 ms and 1044 ms to completely load and render after the start of navigation to the web page.

Broadly, the pipeline developed to produce Ideogram annotation data works as follows:

1. Get data for an SRR accession from NCBI SRA ( National Center for Biotechnology Information: Sequence Read Archive).
2. Count reads for each gene and normalize expression values to TPM units ( Wagner *et al.*, 2012)
3. Get coordinates and type for each gene from a GFF file in the NCBI *Homo sapiens* Annotation Release
4. Format coordinates and TPM values for each gene into JSON used by Ideogram.js

The data pipeline exists in two parts: one for data in SRA and one for data in GEO.

The tool reads a list of SRR accession numbers ( National Center for Biotechnology Information, 2011; National Center for Biotechnology Information: SRA Handbook (2011)) and identifies the ones that have alignment. It then retrieves the genome reference used for the creation of the BAM/SAM file to download the gene annotation for quantification. Only genome assemblies GRCh37 (GCA_000001405.1) and GRCh38 (GCA_000001405.15) are supported, and the annotations used for each of them are NCBI *Homo sapiens* Annotation Release 105 and 107, respectively ( National Center for Biotechnology Information, 2013; National Center for Biotechnology Information, 2015).

Alternatively, the tool can read a BAM/SAM file in case of local files. In one single command, the tool quantifies gene expression using HTSeq-count version 0.6.1p1 ( Anders *et al.*, 2015) after sam-dump version 1.3 ( National Center for Biotechnology Information, 2011). To avoid possible errors due to non-standard SAM files, our filtering steps in the middle sort the BAM file and keep only properly paired reads. The output from HTSeq-count is a tabular file, where the first column is the gene symbol and the second is the read counts. Finally, we normalize the expression by the length of the mature transcript using the longest transcript as the size of the gene.

After obtaining TPM values for each gene’s expression level (Step 2) as described above, the next step in the pipeline parses genomic coordinates (chromosome name, start and stop) and gene type (e.g. mRNA, ncRNA) from a GFF file in the NCBI *Homo sapiens* Annotation Release. These data are combined with each gene’s TPM value, formatted into a compressed JSON structure, and written to a file containing symbols, genomic coordinates, expression levels and gene types for every human gene. This file, e.g. SRR562646.json, represents the final output of the RNA-Seq Viewer data pipeline, and contains all the data used by the fast client-side faceted search in Ideogram.js.

## Results

**Figure 1.**
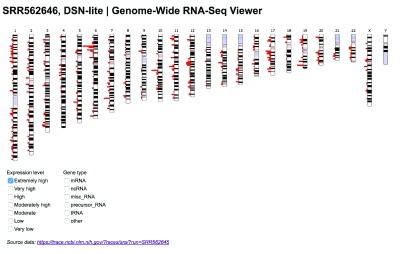
RNA-seq data for all chromosomes can be filtered on demand and viewed en masse.

**Figure 2.**
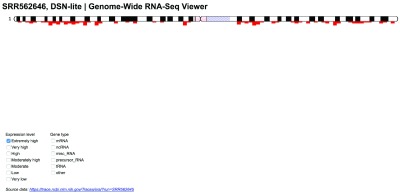
RNA-seq data for a single chromosome can also be filtered and viewed.

The resulting RNA-Seq Viewer web application prototype was demonstrated at the conclusion of the three-day hackathon at Brandeis University. The application provides an interactive data visualization in which users can filter genes by expression level and gene type across the entire human genome ( Figure 1) or within a single chromosome ( Figure 2).

## Discussion

The RNA-Seq Viewer prototype demonstrates a pipeline for transforming aligned RNA-seq data from SRA into a format used for genome-wide visualization.

Next steps for this data pipeline include supporting RNA-seq alignment and normalization when using multiple samples, such as from different tissues. Filters for those different tissues could also be added as filters in the display. The resulting genome-wide visualizations could then be embedded in genome browsers, e.g. NCBI Genome Data Viewer ( National Center for Biotechnology Information: Genome Data Viewer), or any genomics-oriented application that supports HTML, CSS, and JavaScript.

The prototype implemented in the hackathon only supports RNA-seq datasets from SRA that are already aligned to a reference genome, e.g. GRCh37 or GRCh38. Salmon ( Patro *et al.*, 2015) and Kallisto ( Bray *et al.*, 2016) are two popular alignment programs that could be used for this task. Both alignment programs can generate gene expression, with low memory and CPU requirements.

## Software availability

Latest source code: https://github.com/NCBI-Hackathons/rnaseqview

Archived source code as at the time of publication: https://dx.doi.org/10.5281/zenodo.377055 ( Weitz *et al.*, 2017)

License: CC0 1.0 Universal

## References

[ref-1] Anders S Pyl PT Huber W: HTSeq--a Python framework to work with high-throughput sequencing data. *Bioinformatics.* 2015;31(2):166–9. 10.1093/bioinformatics/btu638 25260700 PMC4287950

[ref-2] Barrett T Wilhite SE Ledoux P: NCBI GEO: archive for functional genomics data sets--update. *Nucleic Acids Res.* 2013;41(Database issue):D991–5. 10.1093/nar/gks1193 23193258 PMC3531084

[ref-3] Basques K Kearney M: Chrome Developer Tools: Network Panel Overview [Online].2016; [Accessed: 26 September 2016]. Reference Source

[ref-4] Basques K Kearney M: Chrome Developer Tools: Network Panel Overview [Online].2016; [Accessed: 26 September 2016]. Reference Source

[ref-5] Belson D Thompson J Sun J: Q4 2015 State of the Internet Report. *Akamai Technologies.* 2016;8(4). Reference Source

[ref-6] Bostock M Ogievetsky V Heer J: D ^3^: Data-Driven Documents. *IEEE Trans Vis Comput Graph.* 2011;17(12):2301–2309. 10.1109/TVCG.2011.185 22034350

[ref-7] Bray NL Pimentel H Melsted P: Near-optimal probabilistic RNA-seq quantification. *Nat Biotechnol.* 2016;34(5):525–527. 10.1038/nbt.3519 27043002

[ref-8] Broad Institute: Lightweight html5 version of the Integrative Genomics Viewer [Online].2014; [Accessed: 26 September 2016]. Reference Source

[ref-9] Kent WJ Sugnet CW Furey TS: The human genome browser at UCSC. *Genome Res.* 2002;12(6):996–1006. 10.1101/gr.229102 12045153 PMC186604

[ref-10] Kodama Y Shumway M Leinonen R: The Sequence Read Archive: explosive growth of sequencing data. *Nucleic Acids Res.* 2012;40(Database issue):D54–6. 10.1093/nar/gkr854 22009675 PMC3245110

[ref-11] National Center for Biotechnology Information: Genome Data Viewer [Online].2016; [Accessed: 26 September 2016]. Reference Source

[ref-13] National Center for Biotechnology Information: SRA Handbook [Online].2011; [Accessed: 26 September 2016]. Reference Source

[ref-14] National Center for Biotechnology Information Sequence Read Archive Run Browser [Online]: GSM999527: DSN-lite; Homo sapiens; RNA-Seq (SRR562645).[Accessed: 14 February 2017]. Reference Source

[ref-15] National Center for Biotechnology Information: Using the SRA Toolkit to convert .sra files into other formats.In *SRA Knowledge Base* 2011; [Accessed: 26 September 2016]. Reference Source

[ref-16] National Center for Biotechnology Information: Homo sapiens Annotation Release 105 [Online].2013; [Accessed: 26 September 2016]. Reference Source

[ref-17] National Center for Biotechnology Information: Homo sapiens Annotation Release 107 [Online].2015; [Accessed: 26 September 2016]. Reference Source

[ref-18] Parker CC Gopalakrishnan S Carbonetto P: Genome-wide association study of behavioral, physiological and gene expression traits in outbred CFW mice. *Nat Genet.* 2016;48(8):919–926. 10.1038/ng.3609 27376237 PMC4963286

[ref-19] Patro R Duggal G Kingsford C: Salmon provides accurate, fast, and bias-aware transcript expression estimates using dual-phase inference. *BioRxiv.* 2015 10.1101/021592

[ref-20] Square Inc: Crossfilter [Online].2012; [Accessed: 26 September 2016]. Reference Source

[ref-21] Wagner GP Kin K Lynch VJ: Measurement of mRNA abundance using RNA-seq data: RPKM measure is inconsistent among samples. *Theory Biosci.* 2012;131(4):281–285. 10.1007/s12064-012-0162-3 22872506

[ref-23] Weitz EM: Ideogram [Online].2015; [Accessed: 26 September 2016]. Reference Source

[ref-24] Weitz EM Pantano L Zhu J: NCBI-Hackathons/rnaseqview 1.1. *Zenodo.* 2017 Data Source

