# Supplementary material for: Viewing RNA-seq data on the entire human genome
Source: F1000Res. 2017 Apr 28;6:596. [Version 1] doi: 10.12688/f1000research.9762.1 (PMC5605993; doi:10.12688/f1000research.9762.1)
Supplement: Peer review history

# Peer review: Referee response for version 1

**DOI:** 10.5256/f1000research.10524.r25863

**Published:** 2017-09-20

**Authors:** Philip Ewels

**Affiliations:** 1 Department of Biochemistry and Biophysics, Science for Life Laboratory, Stockholm University, Stockholm, 106 91, Sweden

This manuscript does a great job of describing RNA-Seq Viewer, a tool to visualise genome-wide co-ordinate based clustering of gene expression in a sample. It's a neat project and great to see such a well polished product coming out of a hackathon. Such a responsive tool is impressive, and the user interface is simple to use.

 Minor concerns:

- The abstract says that RNA-Seq Viewer is a *"web application"*. However, to use it users are required to run a series of command line tools to prepare data and then edit a HTML file before getting to the web page. So whilst the tool certainly uses web technologies, I would not say that it's a fully fledged (eg. online only) web application yet. A minor change in wording would be sufficient to clear this up.
- The abstract says that *"The backend data pipeline was developed and deployed on a shared AWS EC2 instance."* - however, this seems to be the only mention of AWS in the manuscript or repository. If the authors mean that they deployed it for a one-off run, I think it's a little misleading (my assumption was that it is running as a service on AWS for anyone to use).
  - Additional documentation as to how users can use AWS to run the tool would also be useful.
- There are example reports in the GitHub repository, but they're not mentioned anywhere that I can see. It would be nice if the readme clearly pointed towards these in the introduction with links using http://rawgit.com so that they can be loaded directly.

Other than this, I think that the manuscript fairly describes the project. I'd love to see the additions that the authors propose at the end (support for multiple samples and use with a wider range of input data) and hope that the manuscript may get a future revision with such additional features!

 I see that another reviewer mentions the generic name - I agree that 'RNAideogram' or something else may be a little more specific and useful!

I have read this submission. I believe that I have an appropriate level of expertise to confirm that it is of an acceptable scientific standard.

# Peer review: Referee response for version 1

**DOI:** 10.5256/f1000research.10524.r25864

**Published:** 2017-09-18

**Authors:** Christopher J. Fields

**Affiliations:** 1 High-Performance Biological Computing Group, Roy J. Carver Biotechnology Centre, University of Illinois at Urbana–Champaign, Urbana, IL, USA

The authors have succeeded in integrating multiple disparate software resources into a useful and well-motivated tool, RNA-Seq Viewer, that was largely put together within a three-day hackathon, which is even more impressive and speaks to the strengths of hackathons in general, particularly when there is a clear motivation and goal in mind that play to the strengths of everyone involved. I'm particularly happy to see that NCBI is more actively engaging the open-source community though organization of workshops and events such as this.

 One key item: I couldn't find an online example, if there is one available this would be very useful as a live demo and would be particularly useful in garnering feedback, including possible directions for future development.

 I should note: there are already a few 'RNA-Seq Viewer' tools out there, not sure if you would need to change the name (if so please no horrible backronyms):

 https://www.ncbi.nlm.nih.gov/pmc/articles/PMC5320319/

 http://bioinfo.au.tsinghua.edu.cn/software/RNAseqViewer/

I have read this submission. I believe that I have an appropriate level of expertise to confirm that it is of an acceptable scientific standard.

# Peer review: Referee response for version 1

**DOI:** 10.5256/f1000research.10524.r22368

**Published:** 2017-05-08

**Authors:** Chase A. Miller

**Affiliations:** 1 Department of Human Genetics, University of Utah, Salt Lake City, UT, USA

The RNA-Seq Viewer tool is well motivated and clearly described in this article. RNA-Seq Viewer does a good job of combining data from multiple public sources into a single coherent visualization and interface. There is a great need for more tools like this that make use of the huge amounts of public genomic data available.

 I found an online example of RNA-Seq Viewer here. It would be very useful if this link was included in the Abstract so that potential users can quickly try out the tool.

 Although beyond the scope of a 3-day hackathon, in the future it would be valuable to turn this tool into a fully hosted web app so that no download or command line knowledge would be required.

I have read this submission. I believe that I have an appropriate level of expertise to confirm that it is of an acceptable scientific standard.
